# The effects of age, size, and cage complexity on the behaviour of farmed female chinchillas (*Chinchilla lanigera*)

**DOI:** 10.1038/s41598-023-32516-5

**Published:** 2023-04-14

**Authors:** Stanisław Łapiński, Piotr Niedbała, Katarzyna Markowska, Agnieszka Rutkowska, Marcin W. Lis

**Affiliations:** 1 Department of Zoology and Animal Welfare, University of Agriculture in Krakow, Al. Mickiewicza 21, 31-120 Kraków, Poland; 2 Department of Animal Reproduction Anatomy and Genomics, University of Agriculture in Krakow, Al. Mickiewicza 21, 31-120 Kraków, Poland; 3 Department of Applied Mathematics, University of Agriculture in Krakow, Al. Mickiewicza 21, 31-120 Kraków, Poland

**Keywords:** Animal behaviour, Behavioural methods

## Abstract

Even though chinchillas have been farmed for a century, there are not many studies concerning their behaviour in captivity or their optimal housing conditions, both of which are important factors in the assessment of their welfare. This study aimed to evaluate the effect of different cage types on chinchillas’ behaviour and their reactions towards humans. Female chinchillas (n = 12) were kept in three types of cages: standard with a wire floor (S); standard with a deep litter floor of shavings (SR); and enlarged with a deep litter floor of shavings (LR). Animals spent 11 weeks in each type of cage. The chinchillas’ reactions toward humans were observed via intruder test. Ethograms were prepared based on round-the-clock video recordings. The activity of the chinchillas was compared, taking into account the different cage types and the animals’ varying responses to the hand test. The generalized ordered logistic regression model was used to ascertain whether cage type has an effect on a chinchilla’s behaviour towards humans. To compare the time distribution of various activity between chinchillas, the non-parametric Scheirer–Ray–Hare test was used. Animals kept in LR cages presented significantly less timid reactions in comparison to those kept in S and SR cages. The chinchillas spent most of their time resting (68% of the day), in locomotion (23%), and eating or drinking (8%); they spent only 1% on grooming behaviour. Cage enrichment generally reduced the fear of humans. However, the average chinchilla response to the hand test was classified in each type of cage as “cautious”. Analyses of the ethograms indicated that the chinchillas were active mostly during the dark stage of the day. In conclusion, the larger cage size and its enrichment (particularly litter) reduced the fearfulness and passivity of the animals, which could be evidence of better welfare conditions.

## Introduction

Under the Five Domains Model^1^, current animal welfare science takes into account nutrition, environment, health, behavioural interactions, and mental state. One aspect of the physical environment is the housing system, which can have a positive or negative effect on animals’ mental state^1^. In addition to the importance of animal housing conditions and animal health, these recommendations also emphasize the expression of normal behavioural patterns. They indicate that it is important to take abnormal behaviours (e.g. excessive aggression or stereotypes) into consideration in animal welfare assessments^2^. These principles underpin the EU-founded Welfare Quality^®^ project, which is the basis of farm animal welfare scoring. On this basis, the European Fur Breeders Association prepared on-farm welfare-assessment protocols for farmed fur animals (foxes and minks)^3–5^. Such detailed protocols have not yet been developed for the chinchilla^6^.

The natural habitat of the long-tailed chinchilla (*Chinchilla lanigera*, Bennne, 1829) covers barren, arid and rugged areas of transverse mountain chains in north-central Chile. These animals are nocturnal and live in herds; however, there is relatively little information about their natural biology (i.e. social behaviour, predators, etc.). As a fur animal, this species has been threatened for many years by hunting and poaching. The wild chinchilla population is continuing to decline due to human activities, such as the pet trade, grazing by cattle and goats, mining, firewood extraction, and El Niño events^7^. Currently, the wild population is estimated at 5350 mature individuals^7^, the distribution of which is restricted to only a few small and fragmented colonies^8^. Therefore, the wild long-tailed chinchilla is endangered under criteria B2ab(i,ii,iii) and was added to the IUCN Red List of Threatened Species in 2015^7^. On the other hand, due to its very valuable fur, the long-tailed chinchilla (*Chinchilla lanigera* Bennett, 1829) has been domesticated since the 1920s and kept as pets and laboratory animals^9^. Although they have been farmed for a century, there are not many studies concerning their behaviour or comprehensively detailing their living conditions^10–13^, both of which are important factors in the assessment of their welfare and degree of domestication. Different guidelines for keeping and caring for chinchillas can be found in the regulations of the European Commission^14^, national regulations of particular countries, and suggestions from animal rights organizations or breeders' associations Furthermore, there are important disagreements between the different guidelines. For example, the Polish National Chinchilla Breeders Association recommends cages with netting or a solid floor with minimum dimensions (width, length, and height) of 0.40 m × 0.45 m × 0.34 m (0.06 m^3^)^15^, the Canadian Standard Guidelines for the Operation of Chinchilla Ranches^16^ require 2200 cubic inches (approx. 0.04 m^3^) for each animal, while the German Veterinary Association for Animal Welfare^17^ advises the use of group housing systems with a minimum volume of 3 m^3^ for two animals (min. 0.5 wide and 1.5 m high) and at least 0.5 m^3^ for every additional animal.

In addition to the cage dimensions, the type of flooring also greatly influences the natural behaviours animals exhibit, such as scratching and dust bathing, when in contact with bedding material^18–20^. It was found that adding various substrates and structural enrichment to cages (shelves, wooden blocks/sticks, deep litter, etc.) enriches this environment, which reduces animals’ undesirable behaviour, such as chinchillas’ fur-chewing^12^ and fear and aggressive reactions in farmed foxes^21^. Environmental enrichment can improve both the physiological and psychological welfare of captive animals, which can be assessed by noting the increased expression of natural behaviour and decreased expression of abnormal behaviours. However, a key factor contributing to how a captive animal interacts with its environment is its relationship with humans^22^. A study in mice kept in standard cages has shown impaired brain development, abnormal repetitive behaviours (stereotypies), and an anxious behavioural profile, all of which can be lessened by making the cage environment more stimulating^23^. Additionally, cage enrichment (e.g. gnawing objects for foxes) allows farm animals to relieve stress more easily, which may improve the animals’ responses to humans^21^.

Farm animals' negative reactions toward humans, which can be caused by limited contact with humans (e.g. only at weaning or vaccinations)^24^, have serious economic, practical, and welfare implications^25,26^.

The most common method of testing fur animals’ reactions to humans is the “stick test”^27^. Based on an animal's immediate reaction to a wooden spatula being inserted into the cage through the mesh, individuals can be categorized as fearful, curious (sometimes referred to as “confident”) or aggressive. This test is also recommended in mink and fox welfare assessment protocols^4,5^. However, “Trapezov’s hand test” is recommended as a more sensitive alternative. In this behavioural test, the cage lid is opened, a gloved hand is slipped in, and the mink is touched if possible^27,28^. On this basis, it can be hypothesized that the size and complexity of chinchilla cages influence their behaviour and responses to humans. Therefore, the study objective was to verify this hypothesis and evaluate the effect of housing conditions on the human-animal relationship. This could help to improve chinchillas’ welfare and provide valuable information on the behaviour this species.

## Materials and methods

### Animals, housing, and management

The reported experiment was approved by the First Local Ethical Committee on Animal Testing at Jagiellonian University in Kraków (Licence no. 45/2014), and all methods and animal treatment used in the study were performed according to the guidelines and regulations stated in Directive 2010/63/EU on the protection of animals used for scientific purposes and the Act 2015/266 of the Republic of Poland on the protection of animals used for scientific or educational purposes. The study reported in the manuscript follows the recommendations in the ARRIVE guidelines.

The domestic chinchillas (standard colour, sexually mature, virgin, unrelated females; eight months old; initial body weight 593 ± 53.3 g; n = 12) were obtained one month before starting the study from a commercial breeding farm (“Raba” Chinchilla Breeding Farm, Myślenice, Poland). They were taken to the chinchilla breeding facility of the University of Agriculture in Kraków, where they were quarantined for four weeks in the same housing system as used at commercial chinchilla farms. Chinchilla females were housed individually in stainless-steel cages (0.40 m width × 0.50 m length × 0.35 m height) with a wire floor. Throughout the study, the animals were exposed to a controlled temperature in the range of 18–22 °C and a photoperiod of 14.5 h light (total natural and artificial)/9.5 h dark.

**Figure 1:**
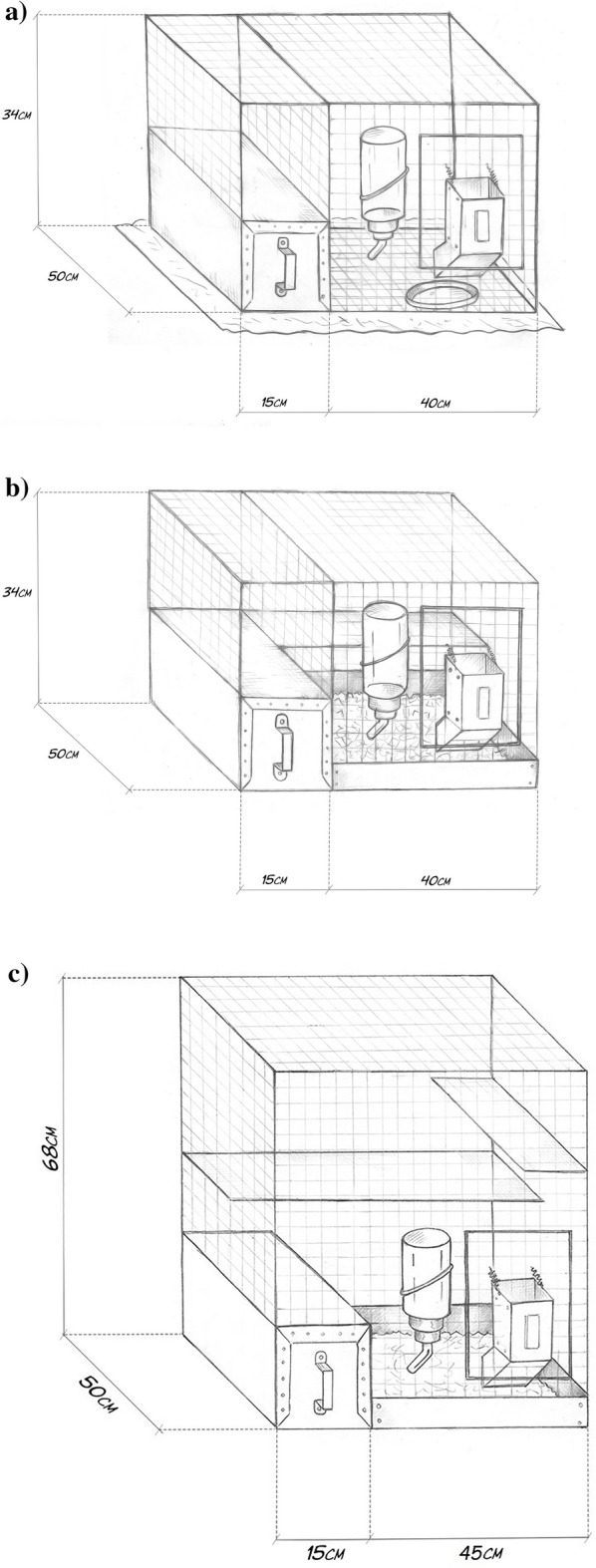
The chinchilla housing types used in the research: (**a**) standard cage (S) with a wire floor, equipped with a ceramic plate under the feeder to reduce the loss of fodder. (**b**) enriched standard cage (SR) with a deep litter floor of shavings and a grid platform on the rear wall. (**c**) enlarged and enriched cage (LR) with a deep litter floor of shavings and two grid platforms on the rear and side walls.

The animals were randomly divided into three groups (four animals per group) and assigned to three experimental conditions (cage type); all cages were equipped with a sand bath and a wooden block:

1. Standard cage with a wire floor (standard, S; 0.40 m width × 0.50 m length × 0.34 m height, Fig. 1a), equipped with a ceramic plate under the feeder to reduce the loss of fodder;
2. Standard cage with a deep litter floor of shavings and a grid platform on the rear wall (enriched standard, SR; 0.40 m width × 0.50 m length × 0.34 m height; Fig. 1b);
3. Enlarged cage with a deep litter floor of shavings and two grid platforms on the rear and side walls (larger and enriched, LR; length 0.60 m × width 0.50 m × height 0.68 m; Fig. 1c).

Each type of cage was placed in one row.

The experiment was carried out between June and March in three blocks of 11 weeks each (A: June–September; B: October–December; C: January–March). Each block consisted of one week of acclimatization and ten weeks of observing the animal in a given cage type). After each block, the same animal was transferred to the next cage type, according to a design of the experiment (Table 1), which allowed for 12 repetitions for each block, so a total of 36 repetitions. The environmental conditions were constant throughout the whole study. In order to reduce individual differences, all animals in the study were already accustomed to handling, and they were treated the same as in standard commercial settings.

**Table 1:** Scheme of the experiment.

Block of experiment	Cage type	Animal			
A (Jun.-Sep.)	S	U4	U36	U26	U28
	SR	U22	U38	U108	U174
	LR	U42	U60	U114	U170
B (Oct.-Dec.)	S	U42	U60	U114	U170
	SR	U4	U36	U26	U28
	LR	U22	U38	U108	U174
C (Jan.-Mar.)	S	U22	U38	U108	U174
	SR	U42	U60	U114	U170
	LR	U4	U36	U26	U28

Distribution of animals in various types of cages.

Cage category designations: S—standard cage with a wire floor; SR—enriched standard cage with a deep litter floor and shelf; LR—enlarged cage with a deep litter floor and shelf.

### Behavioural study

#### Hand test

A modified version of Trapezov’s hand test^27–29^ (with modifications for this study), hereafter “the hand test”, was used to categorize the responses of chinchillas to the intrusion of the researcher’s hand into their cages. To this end, before starting the experiment the researcher (the same person who tested the animals during the experiment) performed preliminary tests on 40 other animals to identify behavioural patterns and develop a rating scale. To prevent the habituation of the animals, the researcher did not work with these chinchillas regularly but only visited the breeding facility occasionally during testing. When performing the test, the researcher put one hand on the open front of the cage and moved it slowly inside. The reaction of the chinchillas to handling attempts was recorded according to a five-point scale (Table 2). Test duration was about 15–30 s, depending on how much the animal interacted with the researcher. The test was carried out in each cage for each animal once a week for the 8 months of the study with a total 30 repetitions per animal. The mean scores of this test were used to classify the animals’ responses: 1.00–1.80 = confident; 1.81–2.60 = cautious; 2.61–3.40 = timid; 3.41–4.20 = nervous; 4.21–5.00 = aggressive.

**Table 2:** Scores of the hand test used to categorize the responses of chinchillas to human intrusion into the cages.

Score	Description
1	Chinchilla is not afraid, sniffs the hand, approaches with interest, allows its head and back to be stroked
2	Chinchilla explores the hand at a distance (no physical contact), approaches but withdraws, does not allow itself to be touched
3	Chinchilla makes warning calls (barks), moves away from the hand, runs around the cage
4	Chinchilla takes flight around the cage and screams, often stands on two paws and attempts to spray the person with urine
5	Before opening the cage, chinchilla makes warning calls, takes flight around the cage, tries to bite

#### Ethogram

The maintenance behaviours were classified and analysed in terms of duration (behavioural states) according to Franchi et al.^13^, with a modification for this study (Table 3). The continuous behavioural observations of the chinchillas were simultaneously carried out using three infrared surveillance charge-coupled device (CDD) cameras (model AT TI560E; one camera was focused on the animals in four cages of one type) and recorded with a time-lapse digital recorder for the last five days of each animal’s residence in a particular cage type. After the observation period, the video recordings (1080 h of recording) were analysed by the same person using General_RECPlayer v1.8 software. The duration of each behaviour was individually documented by the same observer. All observed behaviours were noted in the research protocol with an accuracy of ± 1 min. The obtained data made it possible to create an ethogram of female chinchillas kept in captivity.

**Table 3:** Ethogram of maintenance behaviours observed in chinchillas kept in farm conditions according to Franchi et al. (2016), with a modification for this study.

Category	Behavioural patterns	Definition
Resting (R)	Sleeping, sitting, lying down;	Subject is not moving, lying on its side or sitting motionless with its paws tucked up and lowered head
Moderate locomotion (L1)	Crawling, walking, exploration	Subject is awake, moves around the cage, makes small, single jumps and stops, slowly walks, looks around, sniffs the surroundings and elements of the cage
High locomotion (L2)	Climbing, jumping, running	Subject runs, climbs the walls of the cage, moves vigorously jumping, bouncing with its paws also against the vertical walls of the cage, stops for not more than 10–15 s
Eating pellets (FP)	Investigating feed, eating pellets	Subject approaches the feeder, rummages in the pan or takes a feed pellet in the front paws and begins to eat it
Eating hay (FH)	Investigating feed, eating hay	Subject walks up to the hay rack, picks up a blade and begins to eat
Drinking (FW)	Investigating the drinking outlet, drinking water	Subject approaches the water bottle and begins drinking
Self-directed (GR)	Grooming, shaking, face washing, dust bath	Subject is cleaning its own fur and body (head area, torso, anogenital area, tail) by means of grooming-related behaviours such as licking, scratching, nibbling

### Statistical analyses

The generalized ordered logistic regression (GOLR) model was used to assess the effect of different cage types on the chinchilla’s behaviour^30,31^. The model was selected because of the ordered response of the chinchillas to the hand test experiment. The reference cage was S and the reference block was A. The scheme made it possible to assess the change of the chinchilla’s response due to the move from the ‘worst’ to the ‘best’ cage and from the first to the second and third block. Because measurements were repeated with the same chinchillas, random effects were included in the model, taking into account the specific random effect that each animal has,

${\pi }_{ij}$ is the probability of being in category $j$ assuming that the explanatory variable is $i$. In this case, the reference category is numbered $M$. In the GOLR model, the logarithm of the odds ratio to be in category $j$ compared to the reference category $M$ equals

1 $$\mathrm{log}\frac{{\pi }_{ij}}{{\pi }_{iM}}={\alpha }_{j}+{\beta }_{j}^{T}{X}_{i}$$

for $j\in \left\{1, ... , M-1\right\}$, and $i$ is the number of the covariate. As we observed four different reactions (scores) to the hand test, the level $M=4$ was taken as the reference category. The possible covariates ${X}_{i}$ are (cage LR, block B), (cage LR, block C), (cage SR, block B), (cage SR, block C). The slopes ${\beta }_{j}$ depend on the response variable in the sense that they can be different for various levels $j$ of the response variable even if the covariates are identical.

Taking into account the different types of cages and the animals’ varying responses to the hand test (confident, cautious, timid, nervous, or aggressive), the activity of the chinchillas was compared using the Scheirer–Ray–Hare test (SRH)—the non-parametric equivalence of the 2-way ANOVA^32^.

### Ethical approval and informed consent

The authors confirm that: (1) the reported experiments were approved by the First Local Ethical Committee on Animal Testing at Jagiellonian University in Kraków (Licence no. 45/2014); (2) all methods and animal treatment used in the study were performed according to the guidelines and regulations stated in Directive 2010/63/EU on the protection of animals used for scientific purposes (http://data.europa.eu/eli/dir/2010/63/oj), and the Act 2015/266 of the Republic of Poland on the protection of animals used for scientific or educational purposes (O.J. 2015 pos. 266).

The study reported in the manuscript follows the recommendations in the ARRIVE guidelines (PLoS Biol 18(7): e3000411. https://doi.org/10.1371/journal.pbio.3000411).

## Results

The estimates of the coefficients in the GOLR model [Eq. (1)] were displayed in Table 4. They show that there was a significant association between log odds and cage LR which implies there was a significant change in points from 4 to 1 when chinchillas were moved from cage S to LR, from 4 to 2 when chinchillas were moved from cage S to cages LR and SR, and from 4 to 3 when chinchillas were moved from cage S to LR. As regards blocks, the change from block A to B resulted in a significant change in points from 4 to 1. The change from 4 to 2 or 3 was only weakly associated with the change from block A to B or C. To sum up, chinchillas moved from cage S to LR exhibited a behavioural change towards humans from nervous to confident/cautious/timid. Being moved from block A to B resulted in the change from nervous to confident.

**Table 4:** Coefficients of the GOLR model [Eq. (1)] reflecting the effect of change of cage type on the chinchilla’s behaviour where the reference cage is S, the reference block is A, ${\pi }_{ij}$ is the probability of being in category j assuming that the explanatory variable is $i$, and the reference category (the score of the hand test) is numbered 4.

Item	Intercept ${\alpha }_{j}$	Slopes ${\beta }_{j}$’s			
		cage LR	cage SR	block B	block C
$\mathrm{log }{\pi }_{i1}/{\pi }_{i4}$	− 3.48 (0.111)	**5.08** (0.000)	0.94 (0.188)	**5.92** (0.006)	3.32 (0.085)
$\mathrm{log }{\pi }_{i2}/{\pi }_{i4}$	− 0.76 (0.528)	**5.37** (0.000)	**1.72** (0.011)	3.44 (0.069)	1.53 (0.350)
$\mathrm{log }{\pi }_{i3}/{\pi }_{i4}$	2.20 (0.102)	**3.24** (0.006)	0.29 (0.655)	− 0.33 (0.880)	− 1.51 (0.429)

Significant slope values at $\alpha =0.05$ are marked in bold and the *P* values are in parentheses.

Averaged chinchilla responses to the hand test were classified in each type of cage as cautious (S: 2.4 ± 1.08; SR: 2.3 ± 0.99; LR: 1.9 ± 0.81). It should be noted that no individual in LR was classified as nervous. However, some individuals reacted strongly to cage change, e.g. U26 become more confident after transfers into SR and LR cages while U42 and U60 became nervous/timid after being moved from LR into S cages.

The chinchillas spent most of their time resting (68% of the day), followed by locomotion (23%), eating, and drinking (8%); they spent 1% of their time on grooming behaviour (Fig. 1). However, the type of cage affected the duration of the chinchillas’ maintenance behaviours. In comparison to cage S, animals housed in SR and LR cages spent more time on “extensive locomotion” but less on resting. Moreover, chinchillas in LR cages spent only ~ 10% as much time eating hay as those in other cage types (Table 5). A comparison of the chinchilla’s activity based on hand test results showed that cautious chinchillas spent more time moving around (locomotion) than animals from other classes (Table 6). However, SRH tests showed that there was no difference between the distribution of time the animals spent in each form of activity (*P* = 0.92) for various cage types.

**Table 5:** Duration (mean ± SD) [minutes] and distribution [% of day] of daily activities of chinchillas in the various types of cages.

Activity	Type of cage						Total	
	S		SR		LR			
	% Day	Minute	% Day	Minute	% Day	Minute	% Day	Minute
R	70.6	1017 ± 121.9	65.6	944 ± 91.3	68.5	986 ± 67.8	68.2	982 ± 99.3
L1	9.7	139 ± 65.5	9.0	130 ± 75.5	9.5	137 ± 48.3	9.4	135 ± 63.1
L2	10.8	156 ± 136.7	14.8	213 ± 111.8	14.6	210 ± 81.0	13.4	193 ± 113.4
EP	3.3	47 ± 26.8	4.7	67 ± 19.9	5.1	73 ± 26.6	4.3	62 ± 26.7
EH	4.3	62 ± 53.8	3.2	46 ± 28.3	0.4	6 ± 6.0	2.6	38 ± 42.0
EW	0.7	10 ± 6.3	1.3	18 ± 9.1	0.6	9 ± 7.2	0.8	12 ± 8.6
GR	0.7	10 ± 6.8	1.5	21.8 ± 19.7	1.4	20 ± 12.8	1.2	17 ± 14.8

Cage category designations: S—standard cage with a wire floor; SR—enriched standard cage with a deep litter floor and shelf; LR—enlarged cage with a deep litter floor and shelf.

Behaviour category designations: R—Resting; L1—Moderate locomotion; L2—High locomotion; EP—Eating pellets; EH—Eating hay; EW—Drinking; GR—Self-directed activities.

**Table 6:** Duration (mean ± SD) [minutes] and distribution [% of day] of daily chinchilla activities classified according to hand test results.

Activity	Animal type								Total	
	Confident		Cautious		Timid		Nervous			
	% Day	Minute	% Day	Minute	% Day	Minute	% Day	Minute	% Day	Minute
R	68.0	979 ± 82.9	65.6	945 ± 77.3	69.2	996 ± 127.9	69.8	1005 ± 84.4	68.2	982 ± 99.3
L1	9.4	135 ± 63.9	6.5	94 ± 64.0	9.8	141 ± 52.8	12.5	180 ± 76.2	9.4	135 ± 63.1
L2	12.2	176 ± 102.9	20.8	300 ± 90.0	12.6	181 ± 117.8	11.5	166 ± 119.2	13.4	193 ± 113.4
EP	5.0	72 ± 28.0	3.9	56 ± 16.4	3.8	55 ± 28.2	3.5	51 ± 7.9	4.3	62 ± 26.7
EH	3.1	44 ± 51.2	1.0	14 ± 20.0	3.1	44 ± 35.4	1.4	20 ± 18.1	2.6	38 ± 42.0
EW	0.8	12 ± 10.0	0.9	13 ± 10.0	0.9	13 ± 6.4	0.6	8 ± 5.3	0.8	12 ± 8.6
GR	1.6	23 ± 18.9	1.3	19 ± 6.2	0.8	11 ± 7.8	0.7	10 ± 6.9	1.2	17 ± 14.8

Behaviour category designations: R—Resting; L1—Moderate locomotion; L2—High locomotion; EP—Eating pellets; EH—Eating hay; EW—Drinking; GR—Self-directed activities.

**Figure 2:**
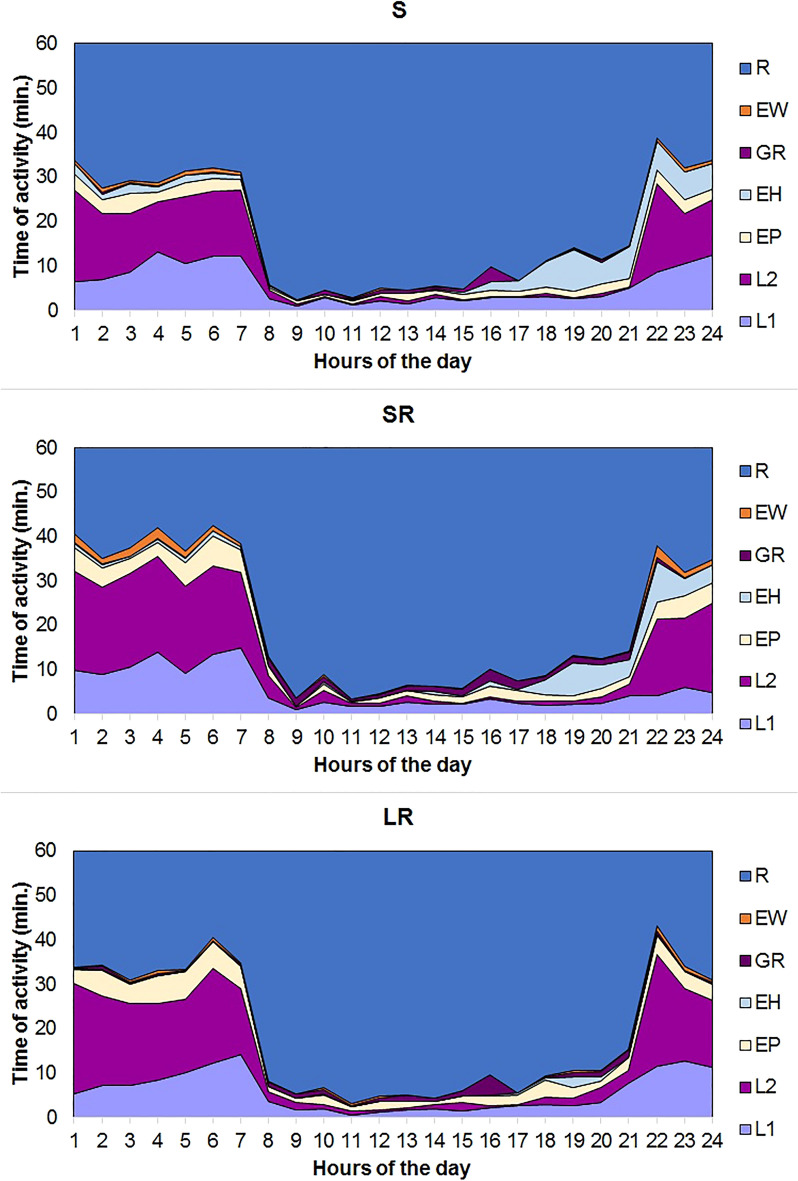
Patterns of the daily behaviour of caged chinchilla females. Cage category designations: S—standard with a wire floor; SR—enriched standard with a deep litter floor and a grid platform; LR—enlarged with a deep litter floor and two grid platforms. Behaviour category designations: R—Resting; L1—Moderate locomotion; L2—High locomotion; EP—Eating pellets; EH—Eating hay; EW—Drinking; GR—Self-directed activities.

Analyses of the ethograms indicated that the chinchillas were active mostly during the dark stage of the day (between 9.30 p.m. and 7 a.m.). Significant regularity of eating behaviour was observed, with the highest hay consumption occurring immediately after the addition of a fresh portion of forage. Grooming activities were only observed between 7:00 a.m. and 9:30 p.m., with the highest intensity at about 4 p.m. (Fig. 2).

## Discussion

One of the most important aspects of farm animals’ welfare is the proper human-animal relationship. Intruder tests/temperament/human intruder tests are used as a non-invasive method to quantify and evaluate animal personalities, and categorize them as fearful or shy and have been widely used for domestic, farm, and even wild animals.

Our preliminary research on chinchillas demonstrated that very few animals showed a pronounced response to the stick test, which made it unsuitable for assessing individual responses. The hand test gave much more unambiguous results and in our opinion it is more useful in research on chinchilla behaviour^33^.

Lapinski et al.’s comparison^34^ of the hand test and the empathic test in assessing the animal (chinchilla)-human relationship indicates that although results of both tests were similar (R = 0.4979), the reaction of the chinchillas in the first one were much more recognizable and clear.

Lack of fear of humans can reduce stress, and confident individuals endure farm conditions better^35^. The results of our experiment show that housing conditions influence chinchillas’ reactions to human intrusion, but the strength of these reactions depends on the individual animal. However, this interpretation requires us to take into account the limited number of studied individuals; the same ones were used for all three cage types. Therefore, the order of cage type could have affected the results. On the other hand, these observations are consistent with those carried out on many other species of farm, laboratory, and zoo animals, e.g. minks^36^, polar foxes^37^, pigs^38^, rats, and mice^39^, as well as non-human primates^40^. For example, enriching the cage environment by adding toys (gnawing objects) caused a significant changing of minks’ personalities, as estimated by empathic tests of the movement from “fearful” towards “confident”, while such a reaction was not observed in “aggressive” ones^12^. Sha et al.^40^, based on observations of captive non-human primates (cotton-top tamarins (*Saguinus oedipus*) and Goeldi’s monkeys (*Callimico goeldii*)), suggest that environmental complexity and/or feeding enrichment) seem to have a larger effect on overall activity levels compared with the effect of larger enclosure sizes on increased species-typical behaviours. Similarly, goats in the enrichment group (afforded enrichments such as food presentation, the use of physical barriers, and the use of elevated areas) had a more excited reaction than the control group, which could be related to their cognitive state thanks to the effect of the enrichment^41^.

The domestication of animals (including chinchillas) involves the selection (intended for animal breeding) of individuals with a mild, balanced character (temperament) that can withstand farm conditions and tolerate human presence^28,42^. These animals generally have better reproductive and health parameters^42–44^. Nevertheless, domesticated animals still show avoidance towards human beings^35,36,45,46^. In the present study, the ‘appropriate behaviour’ of chinchillas, (understood as one of the principles of animal welfare, together with good feeding, good housing, and good health^47,48^) was confirmed by video analysis.

In the course of analysing the video recordings, an additional observation (not included in the research plan) was made that most animals were not only excited and waited at the cage door for the caretaker (not a researcher) at feeding time, but they also started to eat without fear of direct human presence.

In our opinion, this observation might be compared to the results of the feeding test, which is used, for example, to assess responses to human handling in the welfare protocol for foxes^4,21^. Our study also indicates that better responses to human handling are presented by individuals kept in cages with a deep litter floor. In litter cages, the amount of time spent on intensive activity (running) increased at the expense of rest (passivity), which may indicate that litter creates better conditions for movement. Moreover, it was found that chinchillas prefer a quiet, inner corner of the cage for a resting place, and in cages with a mesh floor they use solid areas (ceramic plates) as a resting place.

In the recordings of cages that contained litter, the chinchillas were observed to roll in sawdust. This behaviour (a dust bath) is a natural behaviour observed in chinchillas for fur maintenance. Moreover, Łapiński et al.^12^ noticed that fur-chewing cases decreased in solid-floor cages with litter. These observations support the guidelines that at least 25% of the accommodation floor should be solid for chinchillas^14^. Studies on rabbits confirm the beneficial influence of litter on the welfare of cage-housed animals^49,50^. Straw, which is used as bedding, plays a dual role: it is used as substrate enrichment and as an absorbent for droppings. However, rabbits choose litter so long as it is fresh, but they do not tolerate material spoiled by urine and faeces. Therefore, if regular litter replacement is not possible, then rabbits prefer the cleanliness and the dryness of a wire floor^49,50^. However, the litter material in chinchilla cages is wood shavings, and the production of urine and faeces by this species is lower than that of rabbits; therefore, our study supports the use of bedding in chinchilla caging to improve captive conditions. Our results also indicate that the type of cage (size and floor type) affects the chinchillas’ behaviour. Nevertheless, the cages used as a standard (S and SR) in our experiment have been widely used for decades in chinchilla breeding; they comply with the internal rules of many EU Member States^51^ and seem to have been accepted by most animals in the experiment. It should be noted that the CoE recommendation^14^ proposes that a cage’s minimum cubic capacity for one individual is 0.50 m^3^, which is similar to foxes (0.56 m^3^) and is more than four times bigger than for minks (0.11 m^3^), even though minks and foxes are much larger than chinchillas. The fact that the minimum requirements are met by standard cages is also supported by the fact that in our experiment no stereotypical behaviours were observed in this caging system. However, these behaviours had been observed mostly during the night period in earlier studies by Franchi et al.^13^.

Moreover, chinchillas in LR cages spent less time eating hay than those in other types of cages. One potential explanation for this is that eating hay, apart from its nutritional function, is a form of play (activity), which can compensate for less space and stimuli in S and SR cages. It is known that eating hay, apart from its nutritional function, is a form of play (activity). Animals like to search for food (they choose the tastier parts of hay) even if it is readily available. Hay, straw, or grass satisfy guinea pigs’ and rabbits’ need to chew roughage^39^, which is also observed in chinchillas. In contrast, eating can be a response to boredom in humans^52^. Also, a study on minks indicated that animals without environmental enrichment consume more food than those with environmental enrichment^53^. Chronic inescapable boredom can be can be extremely detrimental to their welfare, and insufficient stimulation can harm neural, cognitive, and behavioural flexibility. Animals in captivity are at particular risk of spatial and temporal monotony, which can have important implications for their welfare^54^.

Analysis of the 24-h video recordings and ethograms generally confirms that chinchillas are crepuscular and nocturnal animals^55^, but Franchi et al.^13^ observed that sleeping and resting are the most frequently observed behaviours for these animals in captivity. In our study, sleeping and resting were mainly manifested in the light period and lasted 52.2 min/h, whilst locomotion lasted 3.7 min/h. In the dark period, locomotion lasted 27.7 min/h, and sleeping and resting lasted 25.2 min/h. However, we must emphasize that changes in the activity pattern occurred rapidly after the light was turned on or off.

In conclusion, the size of the cage and its contents (particularly litter) improves hand test results and seems to stimulate activity, which could be evidence of better welfare of chinchillas.

## Data Availability

The datasets used and/or analysed during the current study are available from the corresponding author upon request.
